# Regulation of Corticosteroidogenic Genes by MicroRNAs

**DOI:** 10.1155/2017/2021903

**Published:** 2017-08-09

**Authors:** Stacy Robertson, Louise A. Diver, Samantha Alvarez-Madrazo, Craig Livie, Ayesha Ejaz, Robert Fraser, John M. Connell, Scott M. MacKenzie, Eleanor Davies

**Affiliations:** ^1^Institute of Cardiovascular and Medical Science, University of Glasgow, Glasgow, UK; ^2^Ninewells Hospital and Medical School, University of Dundee, Dundee, UK

## Abstract

The loss of normal regulation of corticosteroid secretion is important in the development of cardiovascular disease. We previously showed that microRNAs regulate the terminal stages of corticosteroid biosynthesis. Here, we assess microRNA regulation across the whole corticosteroid pathway. Knockdown of microRNA using *Dicer1* siRNA in H295R adrenocortical cells increased levels of *CYP11A1*, *CYP21A1*, and *CYP17A1* mRNA and the secretion of cortisol, corticosterone, 11-deoxycorticosterone, 18-hydroxycorticosterone, and aldosterone. Bioinformatic analysis of genes involved in corticosteroid biosynthesis or metabolism identified many putative microRNA-binding sites, and some were selected for further study. Manipulation of individual microRNA levels demonstrated a direct effect of miR-125a-5p and miR-125b-5p on *CYP11B2* and of miR-320a-3p levels on *CYP11A1* and *CYP17A1* mRNA. Finally, comparison of microRNA expression profiles from human aldosterone-producing adenoma and normal adrenal tissue showed levels of various microRNAs, including miR-125a-5p to be significantly different. This study demonstrates that corticosteroidogenesis is regulated at multiple points by several microRNAs and that certain of these microRNAs are differentially expressed in tumorous adrenal tissue, which may contribute to dysregulation of corticosteroid secretion. These findings provide new insights into the regulation of corticosteroid production and have implications for understanding the pathology of disease states where abnormal hormone secretion is a feature.

## 1. Introduction

Cardiovascular disease is a major cause of mortality and morbidity and therefore a key public health issue. The role of the corticosteroids, aldosterone and cortisol, in the modulation of blood pressure is well known. Their gross excess results in hypertension, and this can be ameliorated by pharmacological or surgical targeting of the production and action of these steroid hormones [1, 2]. Even in essential hypertension, blockade of these hormones lowers blood pressure [3]. However efforts to develop specific aldosterone synthase inhibitors is limited, mostly due to its similarity to 11*β*-hydroxylase. An alternative approach to effectively reduce aldosterone production may be to target regulators of *CYP11B2* expression. Currently, our understanding of the various mechanisms that control corticosteroid biosynthesis in normal and pathological states is incomplete. This must be improved if we are to develop more effective hypertension treatments.

**Figure 1:**
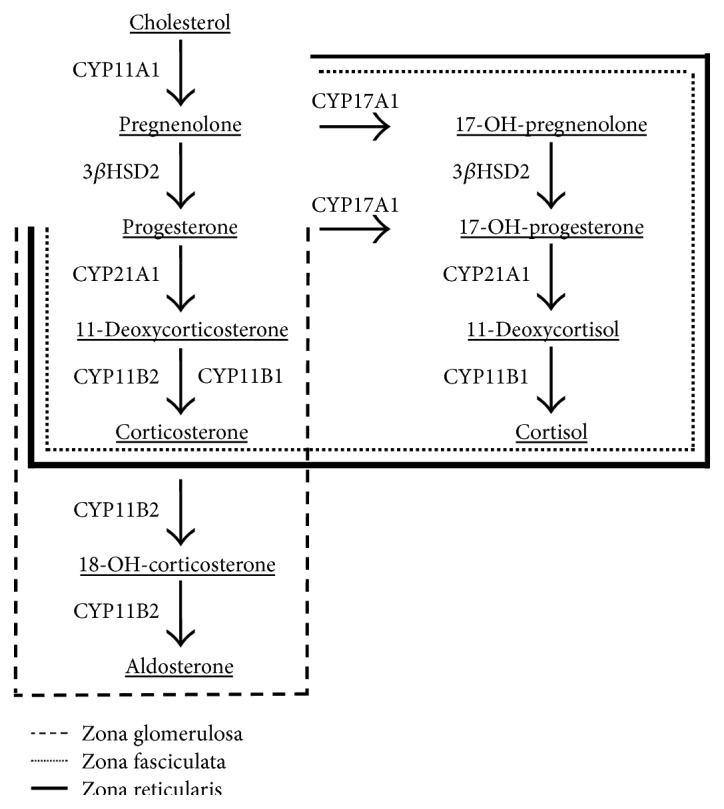
Pathway of corticosteroid synthesis in the adrenal cortex. Dashed lines represent the zone of the adrenal cortex in which each reaction occurs.

Cortisol and aldosterone are the final products of a series of enzyme-controlled reactions that occur in the adrenal cortex. Following transport of cholesterol to the mitochondrion, several cytochrome mixed function oxidases and hydroxysteroid dehydrogenases, each encoded by specific genes, control the integrated series of reactions (Figure 1) culminating in the production of cortisol by 11*β*-hydroxylase (CYP11B1) and aldosterone by aldosterone synthase (CYP11B2) [4]. Cortisol can also be reversibly converted to cortisone by 11*β*-hydroxysteroid dehydrogenases types 1 and 2 (HSD11B1, HSD11B2) [5, 6]. Some intermediate products in these pathways can themselves have deleterious cardiovascular effects, such as the mineralocorticoid, 11-deoxycorticosterone [7].

Expression of corticosteroidogenic enzymes is tightly controlled. This is regulated predominantly at the transcriptional level, but studies by ourselves and others indicate that posttranscriptional modification by microRNA (miRNA) also plays a significant role [8–11]. miRNAs are endogenous small noncoding RNA molecules cleaved from intronically or intergenically located primary miRNA (pri-miR) sequences. Their transcription, in the form of a pri-miR, may be controlled via host gene promoter activity or through a separate miRNA promoter region [12]. They mature via a hairpin structure called a pre-miR, to the final miRNA, this last step being catalysed by the Dicer enzyme [13]. Mature miRNAs are incorporated into the RNA-induced silencing complex (RISC) and use their specific sequence to bind imperfectly to the 3′ untranslated region (UTR) of target mRNA. The RISC contains several endonucleases which can repress target mRNA levels through either translational repression or mRNA degradation. Hence, miRNAs can fine-tune levels of mRNA and contribute to cellular homeostasis [14]. Each step in the corticosteroidogenic pathway represents a potential target for miRNA regulation. Furthermore, a single miRNA is capable of regulating several constituents of a single pathway [15] so that the sum of many small individual effects could result in pronounced changes overall.

Abnormal expression of specific miRNAs can be pathogenic [14, 16]; miRNA levels are altered in adrenal carcinoma and adenoma tissue when compared with normal adrenal tissue [8, 17–23]. However, only a limited number of studies have examined regulation of corticosteroidogenic genes by miRNAs. Previously, we showed that knockdown of *Dicer1* disrupted *CYP11B1* and *CYP11B2* mRNA levels in adrenocortical cells *in vitro*, pointing to a regulatory role for miRNAs [8]. We identified miR-24 as one such miRNA due—at least in part—to its regulatory effects on *CYP11B1* and *CYP11B2* expression [8]. However, the effect of this individual miRNA on total corticosteroid biosynthesis is small and numerous other miRNAs are likely to affect this pathway and probably not only at the terminal points represented by these two genes [8, 10, 11]. The combined regulatory impact of miRNAs simultaneously across all the components of the corticosteroid pathway remains to be assessed.

In this study, we expand the scope of our investigations to encompass all of the genes directly contributing to corticosteroid production, including *CYP11A1* and *CYP17A1* (Figure 1()). We demonstrate that *Dicer1* knockdown by siRNA affects the corticosteroidogenic pathway *in vitro* at numerous points, earlier than the terminal reactions we had previously reported, and that these effects are apparent at both the mRNA and the steroid levels. We then analyse the effects of individual miRNAs on specific transcripts, guided by bioinformatic analysis of gene sequence. Finally, we demonstrate that certain of these corticosteroid-regulating miRNAs are differentially expressed in aldosterone-producing adenoma tissue (APA). We propose that these miRNAs may have a significant pathogenic role and potential diagnostic value.

## 2. Materials and Methods

### 2.1. Cell Culture and Transfection

The H295R human adrenocortical cell line (a gift from Professor William Rainey, Medical College of Georgia, USA) [24] and HeLa cells (European Collection of Animal Cell Cultures, Wiltshire, UK) were maintained as previously described [8] and used between passage numbers 15 and 25. Cells were transfected using siPORT NeoFX Transfection Agent (Applied Biosystems, Warrington, UK) according to the manufacturer's instructions; H295R cells were seeded to a final density of 4.8 × 10^5^ cells/well in 6-well plates and HeLa cells to 8 × 10^4^ cells/well in 24-well plates. Pre-miR™ or Anti-miR™ molecules (miR-125a-5p: product code 12561; miR-125b-5p miR-134-3p 10341; miR-495-3p: 11526; and miR-320a-3p: 11621, Applied Biosystems) were transfected to a final concentration of 50 nM and prevalidated siRNA molecules (Dicer 1A: product code s23755; Dicer 1B: s23756, Applied Biosystems) to a final concentration of 30 nM. Reporter constructs were cotransfected with pEZX construct (500 ng) and either a Pre-miR or an Anti-miR.

### 2.2. RNA Isolation and Real-Time Quantitative Reverse Transcriptase PCR (qRT-PCR)

Total RNA was isolated from H295R cells using the miRNeasy mini kit (QIAGEN, Crawley, UK) according to the manufacturer's instructions. First-strand cDNA was synthesised in a 20 *μ*L volume using the miScript RT kit (QIAGEN) and 200 ng of total RNA. The resulting cDNA was diluted to a final volume of 100 *μ*L of which 2 *μ*L was amplified by qRT-PCR on the ABI PRISM 7900HT apparatus (Applied Biosystems). Reactions used the miScript SYBR Green PCR kit (QIAGEN) to measure mature miRNA-derived cDNA sequences and the Universal ProbeLibrary System (Roche Applied Science, Indianapolis, USA) and ABsolute™ QPCR ROX Mix (Abgene, Epsom, UK) to measure mRNA cDNA using specific primer sequences (Table 1).

### 2.3. Steroid Measurement

Steroids were extracted from cell media using ChemElute cartridges (Varian) and eluted from the cartridge with dichloromethane. The eluates were evaporated to dryness and reconstituted in 10% acetonitrile. Identification and quantification of steroid products was achieved by tandem mass spectrometry using a Varian 1200 L mass spectrometer with a triple quadrupole detector [25].

### 2.4. Identification of the Genomic Location of miRNAs and Prediction of miRNA Targets

The genomic coordinates, strand location, and mRNA transcript length of the human genes encoding corticosteroidogenic enzymes were identified using the Ensembl Genome Browser (release 79, March 2015). These were cross-referenced with known miRNA precursor sequences mapped and stored in the miRBase database (release 21, June 2014). Putative miRNA target sites in the 3′UTRs of corticosteroidogenic genes were identified by four commonly used prediction algorithms (MicroCosm Targets (v.5), microRNA.org (Nov 2010), miRviewer (June 2005), and TargetScan (v. 6.2)).

### 2.5. Reporter Construct Studies

A luciferase reporter construct was purchased from LabOmics (Nivelles, Belgium) to determine miRNA binding to the 3′UTR. The construct (pEZX-B2) contained a pEZX reporter backbone, which comprises a renilla reporter gene coupled to a SV40 viral promoter, a firefly experimental gene coupled to a CMV promoter and the full-length 3′UTR sequence of the *CYP11B2* gene. This was cotransfected with Pre-miR or Anti-miR into HeLa cells; a “no-3′UTR insert” vector (pEZX-C) was used as a control (LabOmics). Firefly and renilla luciferase activity was measured 48 hours posttransfection using the Dual Luciferase Reporter Assay system (Promega, Madison, USA) and a Lumat LB 9507 tube luminometer (Berthold Technologies, Harpenden, UK).

### 2.6. Human Adrenal Gland miRNA Microarray Analysis

Four frozen nondiseased predominantly cortical adrenal tissue samples were obtained from white adult patients undergoing nephrectomy, with full local ethical approval from the University of Birmingham, UK. Four samples of formalin-fixed paraffin-embedded (FFPE) APA tissue were obtained from the University of Glasgow Biobank. Use of tissue in this study was conducted in accordance with the requirements of the Human Tissue Act and with appropriate permission from the local ethical review board. Participants gave informed consent. Total RNA from 40 mg frozen tissue samples or from four 20 *μ*m FFPE tissue sections was prepared using the miRNeasy mini kit (QIAGEN) or the RecoverAll™ Total Nucleic Acid Isolation kit (Applied Biosystems) according to the manufacturer's instructions, respectively. miRNA microarray analyses for 723 miRNAs (miRBase v 10.1) were performed by LC Sciences (Houston, Texas, USA), using 5 *μ*g of the total RNA, as previously described [8]; selected data from this study was previously published [8].

### 2.7. Data Analysis

qRT-PCR results were analysed using the relative quantification method of comparative C_t_ (ΔΔC_t_) [26]. *In vitro* results were analysed using either an unpaired Student's *t*-test or one-sample *t*-test as stated. Statistical analysis was performed using Graph Pad Prism 6.0 software and significance reached when *p* < 0.05. All results are expressed as mean ± standard error of the mean (SEM). Unless stated otherwise, *in vitro* experiments were performed in at least three technical replicates, on three biologically independent occasions (*n* = 3).

## 3. Results

### 3.1. siRNA Knockdown of Dicer1 Increases Levels of Selected Steroidogenic mRNAs and Steroid Secretion in H295R Cells

**Figure 2:**
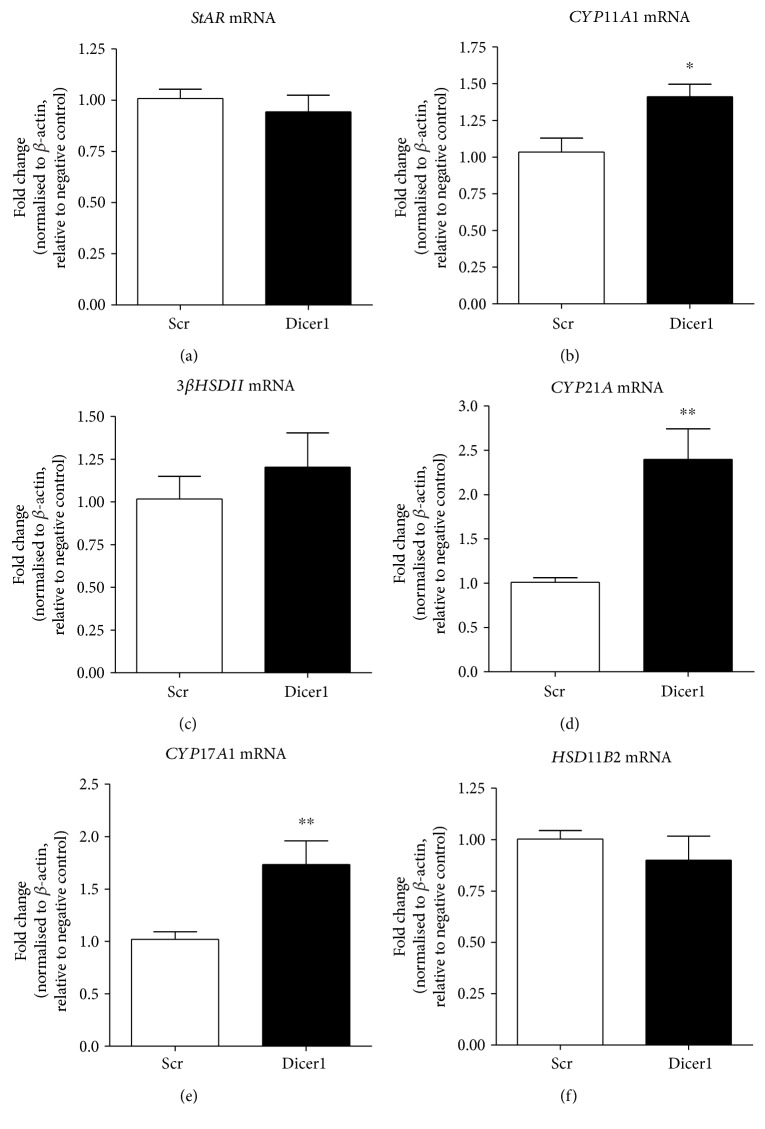
Results from Dicer1 siRNA transfection of H295R adrenocortical cells. Cells were transfected with Dicer1 siRNA or with a scrambled negative-control siRNA (mRNA levels for *StAR* (a), *CYP11A1* (b), *3βHSD11* (c), *CYP21A* (d), *CYP17A1* (e), and *HSD11B2* (f) were analysed 48 hours posttransfection by qRT-PCR). Cycle threshold values were normalised to *β*-actin mRNA and expressed relative to control cell levels. Results represent the mean of three independent biological experiments performed in triplicate ± SEM; ^∗^*p* < 0.05, ^∗∗^*p* < 0.01 compared to scrambled control.

**Figure 3:**
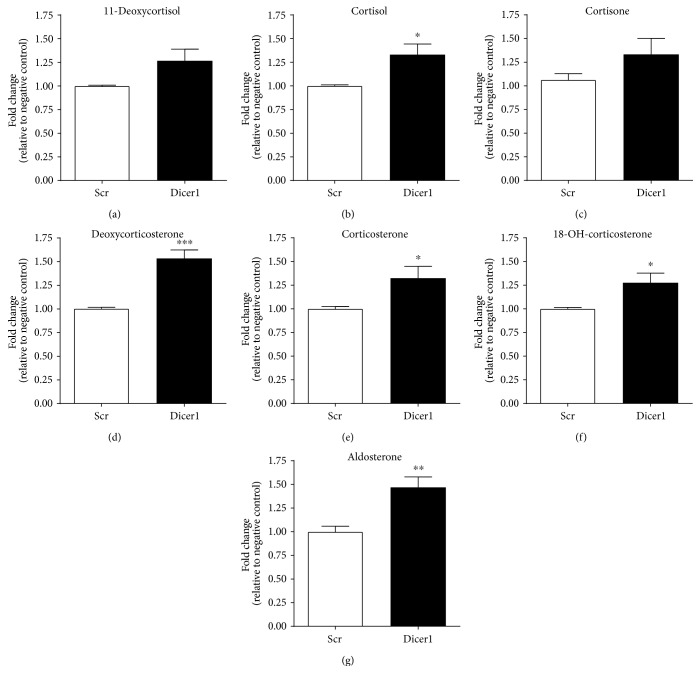
Results from Dicer1 siRNA transfection of H295R adrenocortical cells. Cells were transfected with Dicer1 siRNA or with a scrambled negative-control siRNA. Cortisol-related steroid levels for 11-deoxycortisol (a), cortisol (b), and cortisone (c) and aldosterone-related steroid levels for deoxycorticosterone (d), corticosterone (e), 18-OH-corticosterone (f), and aldosterone (g) were measured 48 hours posttransfection by LC MS:MS. Results represent the mean of three independent biological experiments performed in triplicate ± SEM; ^∗^*p* < 0.05, ^∗∗^*p* < 0.01, and ^∗∗∗^*p* < 0.001 compared to scrambled control.

Significant knockdown of *Dicer1* mRNA by both *Dicer1A* and *Dicer1B* siRNA transfection into the H295R adrenocortical cell line was demonstrated (0.56 ± 0.07-fold and 0.50 ± 0.06-fold, resp.; *p* < 0.001 for each versus scrambled control siRNA) [8]. Total RNA was isolated 48 hours posttransfection from cells transfected with scrambled control siRNA or *Dicer1A* siRNA; levels of mRNAs were measured by real-time qRT-PCR (Figure 2). Significant reduction in *Dicer1* mRNA level did not significantly affect the abundance of *StAR*, *3βHSDII*, or *HSD11B2* mRNAs, but those of the three cytochrome P450-encoding mRNA (*CYP11A1*, *CYP21A1*, and *CYP17A1*) were all significantly increased relative to cells transfected with a scrambled control siRNA (1.41 ± 0.09-fold *p* < 0.05; 2.40 ± 0.34-fold, *p* < 0.01; and 1.73 ± 0.22-fold, *p* < 0.01, resp.). Steroid secretion from H295R cells was measured by LCMS MS:MS 48 hours after transfection with a scrambled control or *Dicer1B* siRNA (Figure 3). Knockdown of *Dicer1* significantly increased secreted levels of cortisol (1.33 ± 0.11-fold; *p* = 0.01), corticosterone (1.32 ± 0.13-fold; *p* = 0.03), 11-deoxycorticosterone (1.53 ± 0.09-fold; *p* < 0.001), 18-hydroxycorticosterone (1.29 ± 0.10-fold; *p* = 0.04), and aldosterone (1.47 ± 0.11-fold; *p* < 0.01) relative to scrambled siRNA transfected cells. Levels of 11-deoxycortisol and cortisone rose but did not achieve statistical significance.

### 3.2. Corticosteroidogenic Genes Contain Putative 3′UTR miRNA Target Sites

Putative miRNA binding sites were identified in the 3′UTR in each of the seven human corticosteroidogenic genes (*CYP11B1*, *CYP11B2*, *CYP11A1*, *CYP17A1*, *CYP21A2*, *HSD3B2*, and *StAR*) and in the two metabolising genes (*HSD11B1*, *HSD11B2*) analysed (Table 2). *CYP11B1* has the longest 3′UTR (2022 base pairs) and contains the highest number of putative miRNA binding sites (390 sites). Conversely, *CYP17A1* has the shortest 3′UTR and the lowest number of predicted miRNA binding sites (59 sites).

### 3.3. The miR-125 Family Regulates CYP11B2 mRNA Expression

**Figure 4:**
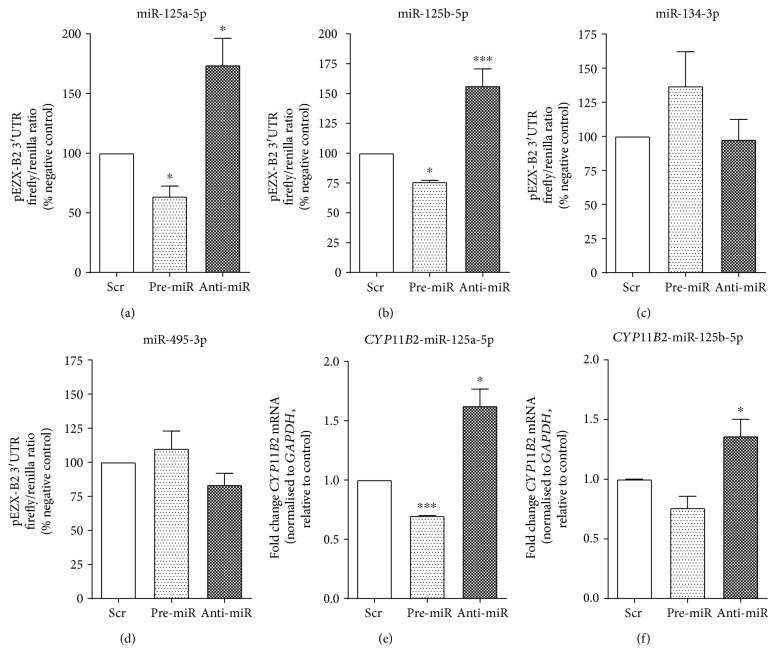
(a–d) Results from HeLa cells transfected with pEZX-B2 3′UTR reporter construct plasmids and either a specific miRNA Pre-miR or Anti-miR, or with a scrambled negative control RNA. Firefly and renilla luciferase luminescence was measured 48 hours posttransfection, and results are presented as firefly reporter gene luminescence normalised for renilla luminescence. H295R cells transfected with either miR-125a-5p (e) or miR-125b-5p (f) Pre-miR, miR-24 Anti-miR, or with a scrambled negative control RNA. *CYP11B2* mRNA was measured 48 hours posttransfection by qRT-PCR, with cycle threshold values normalised to *GAPDH* mRNA and expressed relative to negative control. Results represent the mean of three independent biological experiments performed in triplicate ± SEM; ^∗^*p* < 0.05, ^∗∗∗^*p* < 0.001 compared to negative control.

In order to identify individual miRNAs contributing to the net miRNA effect, as observed under *Dicer1* knockdown, four miRNAs (miR-125a-5p, miR-125b-5p, miR-134-3p, and miR-495-3p) expressed in human adrenal tissue and with putative binding sites in the 3′UTR of *CYP11B2* were selected for further study. Luciferase reporter constructs containing the 3′UTR of *CYP11B2* (pEZX-B2) were transfected into HeLa cells alongside Pre-miR or Anti-miR molecules specific to each of these miRNAs. Levels of luciferase activity were then measured (Figures 4(a), 4(b), 4(c), and 4(d)). Manipulation of miR-134-3p or miR-495-3p levels in this manner did not significantly affect the luciferase activity of the pEZX-B2 reporter construct. However, the presence of active binding sites predicted for miR-125a-5p and miR-125b-5p was confirmed by changes in luciferase activity. Increasing the levels of miR-125a-5p in Pre-miR-transfected cells reduced luciferase activity to 63.78 ± 8.70% (*p* = 0.011) while reduction of miR-125a-5p levels in Anti-miR-transfected cells significantly increased luciferase activity to 173.72 ± 22.54% (*p* = 0.040). Similarly, miR-125b-5p Pre-miR significantly decreased luciferase activity (75.90 ± 1.28%; *p* = 0.033), while its Anti-miR resulted in a significant increase (156.39 ± 14.26%; *p* = 0.017). These responses are consistent with canonical miRNA action.

To determine the direct effect of these miRNAs on *CYP11B2* mRNA expression, Pre-miR or Anti-miR molecules were then transfected into the H295R adrenocortical cell line. Here, miR-125a-5p Pre-miR decreased *CYP11B2* mRNA abundance (0.69 ± 0.002-fold; *p* < 0.0001), while its Anti-miR significantly increased it (1.62 ± 0.014-fold *p* = 0.011; Figure 4(e)). miR-125b-5p Pre-miR transfection reduced *CYP11B2* mRNA to 0.75 ± 0.09 8-fold of control levels, but this did not attain statistical significance (*p* = 0.069). However, miR-125b-5p levels with Anti-miR did significantly increase *CYP11B2* mRNA to 1.36 ± 1 0.04-fold (*p* = 0.041) of control levels (Figure 4(f)).

### 3.4. miR-320a Is a Common Regulator of CYP17A1 and CYP11A1

**Figure 5:**
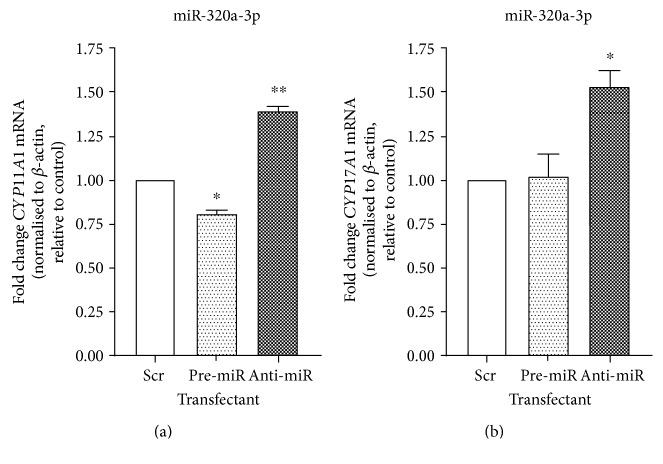
H295R cells transfected with miR-320a-3p Pre-miR, miR-320a-3p Anti-miR, or with a scrambled negative control RNA. *CYP11A1* (a) or *CYP17A1* (b) mRNA was measured 48 hours posttransfection by qRT-PCR, with cycle threshold values normalised to *β*-actin mRNA and expressed relative to negative control. Results represent the mean of three independent biological experiments performed in triplicate ± SEM; ^∗^*p* < 0.05 and ^∗∗^*p* < 0.01 compared to negative control.

Bioinformatic analysis predicted a miRNA binding site for miR-320a-3p in the 3′UTR of two corticosteroidogenic genes: *CYP11A1* and *CYP17A1*. This was tested by transfecting H295R cells with miR-320a-3p Pre-miR and Anti-miR molecules and then measuring mRNA 48 hours posttransfection by qRT-PCR (Figure 5). Raising miR-320a-3p led to significantly decreased *CYP11A1* mRNA (0.81 ± 0.02-fold; *p* < 0.05) but did not significantly affect *CYP17A1* (1.02 ± 0.12-fold). Reduction of miR-320a-3p levels in H295R cells significantly increased both mRNAs: *CYP11A1* (1.40 ± 0.03-fold; *p* < 0.01) and *CYP17A1* (1.53 ± 0.09-fold; *p* < 0.05).

### 3.5. miRNA Expression Differs in Normal Adrenal Tissue and Aldosterone-Producing Adenoma Tissue

**Figure 6:**
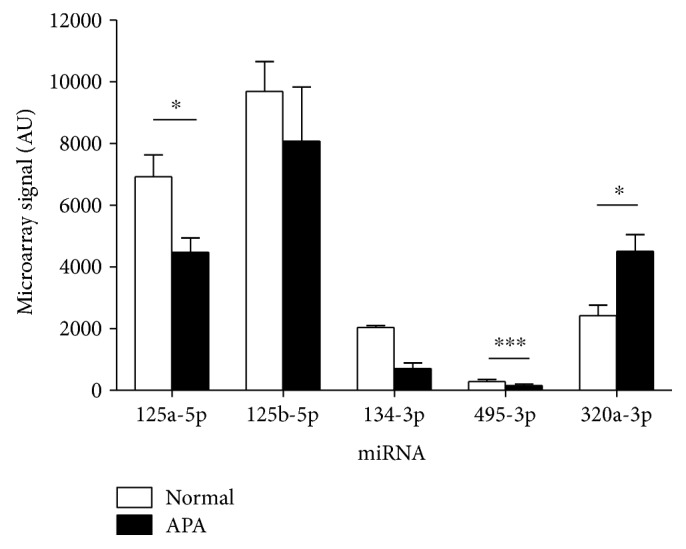
Expression levels of five miRNAs in normal adrenal tissue and APA tissue, as determined by microarray. Data show the normalised microarray signals, with a threshold value of >500 arbitrary units (AU) for at least one tissue type; ^∗^*p* < 0.05, ^∗∗∗^*p* < 0.001.

In a previous study, miRNA expression was assessed by microarray analysis using total RNA samples isolated from four nondiseased human adrenal glands and four human aldosterone-producing adenomas (APAs) [8]. Of the five miRNAs investigated in the current study, two (miR-125a-5p and miR-495-3p) were expressed at significantly lower levels in APA tissue relative to nontumorous tissue; one (miR-320a-3p) was significantly increased in APA tissue and two (miR-125b-5p and miR-134-3p) did not differ significantly between the tissue types (Figure 6).

## 4. Discussion

The *in vitro* investigations conducted during this and our previous study [8] show that general reduction in miRNA levels through knockdown of *Dicer1* significantly increases the abundance of all cytochrome P450-encoding mRNAs in this pathway (i.e., *CYP11A1*, *CYP21A1*, *CYP17A1*, *CYP11B1*, and *CYP11B2*). This confirms that miRNA can exert a net inhibitory effect on the expression of these key corticosteroidogenic genes which is reflected in altered steroid profile. *Dicer1* knockdown increased levels of 11-deoxycortisol, corticosterone, 18-hydroxycorticosterone, cortisol, and aldosterone although the change in 11-deoxycortisol did not achieve statistical significance. The lack of effect on *StAR* expression has already been reported [27]. Interestingly, the dehydrogenase enzymes were not affected; the *HSD11B1* and *HSD11B2* products control the interconversion of cortisol and cortisone in many target tissues, but their mRNA levels were unaffected by *Dicer1* knockdown. Moreover, levels of cortisone were not significantly altered. These two genes and their respective products are not primary products of the adrenal cortex, and while these enzymes do not appear subject to miRNA regulation in H295R cells, other studies suggest miRNAs may regulate *HSD11B1* and *HSD11B2* in other tissues where they have greater functionality [28, 29]. These results indicate that a general inhibition of miRNA synthesis in H295R cells allows increased expression of most, but not all, corticosteroidogenic mRNA transcripts and a rise in steroid products.

Bioinformatic analysis identified putative miRNA target sites in all of the genes tested, although the number of sites predicted at each locus varied markedly depending on the algorithm used. This variability is well known [30], and so it is common to employ multiple algorithms in order to reduce error, as was the case here. These algorithms examine only 3′UTR sequences, and so do not identify possible miRNA binding sites within coding regions or 5′UTR [31, 32]. Therefore, our studies were restricted to miRNA targeting of corticosteroidogenic mRNAs at the 3′UTR. We also used bioinformatic tools to investigate whether the introns of these nine genes harbour any of the 1881 currently mapped human pri-miRNA sequences that occur throughout the genome, but none was found (data not shown). Therefore, any miRNA identified as controlling these genes is not cotranscribed with them.

Since bioinformatic analysis identified miRNA 3′UTR binding sites in all of the genes studied, it is not clear why some genes are apparently refractory, other than to note that predictive algorithms are not perfect, hence the need for experimental verification. Alternatively, structural differences may determine susceptibility; in this study, cytochrome P450 enzymes responded but dehydrogenases did not. Moreover, the 3′UTRs of the enzymes varied widely in their length and number of binding sites they were predicted to contain. Shorter 3′UTRs are thought to be more resistant to miRNA regulation [33].

miR-125a-5p and miR-125b-5p (but not miR-134-3p or miR-495-3p) altered *CYP11B2* expression *in vitro* through direct targeting of its transcript 3′UTR. These two miRNAs belong to the same family, differing by only one nucleotide. However, miR-125a is located on human chromosome 19 in a cluster with miR-99b and let-7c, while miR-125b can originate from either human chromosome 11 (miR-125b-1) or human chromosome 21 (miR-125b-2). They are located intergenically, and miR-125b-2 is close to a gene encoding a long, noncoding RNA (lncRNA). The miR-125 family has been implicated in the regulation of several cell types and has many confirmed targets, including vascular cell cycle-related genes of importance in hypertension [34, 35]. *CYP11A1* and *CYP17A1* are both targets for miR-320a-3p. Overexpression of miR-320-3p did not significantly affect *CYP17A1* mRNA levels but inhibition enhanced levels of both mRNAs; this implies that *CYP17A1* is already under maximal inhibition at the miR-320-3p target site. *CYP17A1* determines relative flow of steroid biosynthesis through the 17-hydroxy and 17-deoxy pathways. An influence here might affect mineralocorticoid-glucocorticoid balance. Like most miRNAs characterised to date, miR-320-3p has been reported to have multiple mRNA targets and regulate different pathways in different cell types. For example, it has been implicated as a tumor suppressor in leukaemia [36] but has also been associated with a detrimental effect in cerebral ischaemia [37].

These results show the potential for several miRNAs to modulate corticosteroidogenesis at the majority of relevant loci. It is not possible to ascertain from these data whether the overall effect is due to several small individual effects or whether action at one locus predominates. We believe there are many more miRNA-mediated effects on this pathway than have been identified thus far, and it may be that the individual miRNA effects we have investigated here are not the most significant. This is borne out by the fact that qRT-PCR analysis of specific miRNA levels in the *Dicer1* knockdown cells showed no significant reduction on the levels of the miRNAs miR-125a-5p, miR-125b-5p, miR-134-3p, miR-320a-3p, and miR-495-3p relative to controls (data not shown). While the *Dicer1* knockdown experiments were not designed with the intention of analysing individual miRNA levels, or powered accordingly, it is tempting to speculate that other miRNAs undergoing a greater dynamic shift as a result of *Dicer1* knockdown will emerge as those most responsible for the observed changes in phenotype. Importantly, if miRNAs are to be incorporated into the existing model of control, more information on the control of their own production is required. For example, it was previously shown that miR-21 levels increase in H295R cells following angiotensin II stimulation [10]. It must also be emphasised that the H295R cell line is derived from neoplastic tissue with an atypical steroid profile [24]. Also, in dispersed cell experiments, the strict zonal constraints on function and gene expression of the intact gland (e.g., the zonally distinct expressions of *CYP11B1* and *CYP11B2*) are absent. Nevertheless, the H295R cell has proved a valuable guide to in vivo adrenal physiology [24].

There is obvious interest in assessing the significance of such miRNA repression in vivo. We compared the expression profiles of miRNAs in normal adrenal and APA tissue and identified differential expression of five miRNAs, in agreement with previous studies [8, 17–23]. miR-125a-5p expression was lower in APA. APA secretes high levels of aldosterone, and the study described above showed *CYP11B2* to be affected by this miRNA. However, its clustered miRNAs were each more abundant in APA tissue than in normal adrenal tissue. Previous studies report reduced *CYP17A1* expression in APA [38]. Our study has shown a high level of miR-320a-3p in the carcinoma-derived H295R cell line and its potency as a *CYP17A1* regulator. miRNA targeting of transcription may be a common feature of this condition.

In summary, by manipulating their levels, we have shown that miRNAs affect the expression of multiple corticosteroidogenic genes and thereby affect the steroid profile at multiple points. Corticosteroidogenic gene sequences include 3′UTR sites that enable their posttranscriptional regulation by specific miRNAs, as demonstrated here for *CYP11B2*, *CYP11A1*, and *CYP17A1*. Importantly, a miRNA which affects aldosterone synthesis and another that affects *CYP17A1* are expressed at different levels in normal adrenal and APA tissue suggesting relevance to this form of adrenal pathology.

## Figures and Tables

**Table 1:** Sequences of primers used in qRT-PCR assays.

Gene	Forward primer	Reverse primer
*β*-Actin	CCAACCGCGAGAAGATGA	CCAGAGGCGTACAGGGATAG
GAPDH	GCTCTCTGCTCCTCCTGTTC	ACGACCAAATCCGTTGACTC
StAR	TACGTGGCTACTCAGCATCG	ACCTGGTTGATGATGCTCTTG
CYP11A1	AGGAGGGGTGGACACGAC	TTGCGTGCCATCTCATACA
CYP21A1	GAGGGCACAGTCATCATTCC	GCTCCAGGAAGCGATCAG
CYP17A1	CTATGCTCATCCCCCACAG	TTGTCCACAGCAAACTCACC
HSD3B2	AGGCCTTCAGACCAGAATTG	CCTCAAGTACAGTCAGCTTGG
CYP11B1	ACTAGGGCCCATTTTCAGGT	GGCAGCATCACACACACC
CYP11B2	GCACCTGCACCTGGAGATG	CACACACCATGCGTGGTCC
HSD11B2	GGGTCAAGGTCAGCATCATC	CACTGACCCACGTTTCTCAC

**Table 2:** Bioinformatic miRNA target site predictions for corticosteroidogenic genes.

Gene	3′UTR length (base pairs)	MicroRNA.org		miRviewer		TargetScan		MicroCosm targets		Unique miRNAs with binding sites
*CYP11B1*	*2022*	151	(142)	4	(4)	436	(333)	33	(30)	**390**
*CYP11B2*	*1428*	89	(83)	10	(10)	323	(253)	6	(6)	**288**
*CYP11A1*	*213*	26	(26)	1	(1)	51	(50)	43	(43)	**68**
*CYP17A1*	*171*	24	(24)	0		35	(34)	23	(22)	**59**
*CYP21A2*	*508*	57	(56)	11	(10)	78	(70)	25	(22)	**134**
*HSD3B2*	*414*	68	(63)	0		73	(68)	46	(44)	**125**
*StAR*	*624*	110	(101)	4	(4)	273	(244)	11	(11)	**285**
*HSD11B1*	*343*	167	(135)	1	(1)	79	(72)	70	(65)	**159**
*HSD11B2*	*550*	82	(78)	4	(4)	116	(104)	33	(31)	**159**

The 3′UTR lengths of these 9 genes were identified using the UCSC Genome Browser Gateway and Ensembl Genome Browser. The number of predicted miRNA target sites from each database is listed, and the number of unique miRNAs predicted to bind each is shown in parentheses. The final column shows the cumulative number of miRNA target sites, with duplicates removed.

## Data Access

All data are included in the manuscript, and requests for additional information should be made to the corresponding author.
